# A nonstop thrill ride from genes to the assembly of the T3SS injectisome

**DOI:** 10.1038/s41467-023-37753-w

**Published:** 2023-04-08

**Authors:** Itzhak Fishov, Sharanya Namboodiri

**Affiliations:** 1 Department of Life Sciences, Ben Gurion University of the Negev, Beer Sheva, Israel; 2 Department of Physics, Ben Gurion University of the Negev, Beer Sheva, Israel

**Keywords:** Bacterial secretion, Bacteriology

## Abstract

The type three secretion system (T3SS) is a membrane-anchored nano-machine utilized by many pathogenic bacteria to inject effector proteins and thus take control of host cells. In a recent article, Kaval et al. reveal a striking colocalization of a T3SS-encoding locus, its transcriptional activators, protein products, and the complete structure at the cell membrane, which they claim provides evidence for a mechanism known as ‘transertion’.

The bacterial type III secretion system (T3SS) is an impressive biological machine known not only for its importance in bacterial virulence^1,2^ but also, due to its evolutionary relationship to the bacterial flagellum, for its role in the negation of the ‘irreducible complexity’ of flagella^3^. In their recent publication^4^, Kaval et al. present new data on the process of expression, production, and assembly of a T3SS (termed T3SS2) in the bacterium *Vibrio parahaemolyticus*. They conclude that the spatial arrangement of this process provides evidence for the hypothetical mechanism of simultaneous transcription, translation, and insertion of integral membrane proteins called transertion. In this comment, we will briefly describe the transertion model’s essence, and then discuss the contribution of Kaval et al. findings to its support.

## Membrane anchoring of the chromosome: transertion model

Chromosome-membrane interaction was suggested probably for the first time by Jacob, Brenner, and Cuzin^5^ aimed to explain the chromosome segregation mechanism. The proposed mechanism contained two major assumptions particularly relevant to this topic: the replicon and the chromosomal origin are attached to the cell envelope, and cell elongation occurs at a specific ‘growth zone’ between the anchored replicons, thus driving them apart to daughter cells. These assumptions inspired numerous investigations. It was demonstrated later that peptidoglycan synthesis takes place roughly homogeneously (or in a large number of sites) along the lateral part of the cell rod^6^ (reviewed in refs. ^7,8^), negating the zonal growth. DNA-membrane interactions, in contrast, were continuously challenged and remain an open question up to now (see ref. ^9^ for a review). Several indications for the existence of DNA-membrane complexes were found in early works^10–13^, but a search for dedicated proteins failed. Specific proteins have been found to interact both with the membrane and DNA, but their function is different, e.g. prevention of division in the nucleoid zone^14^.

**Fig. 1:**
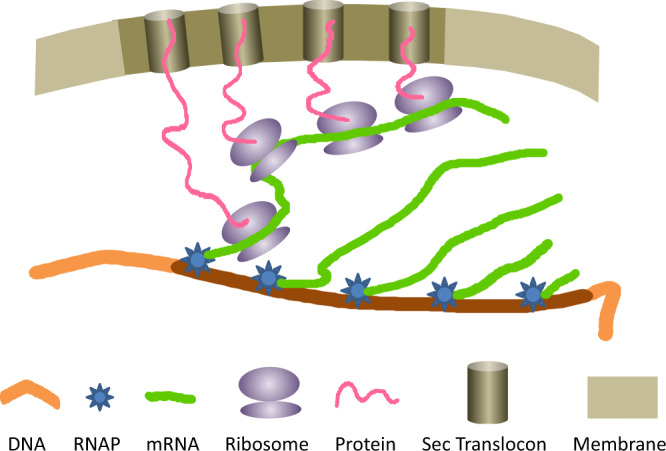
Schematic presentation of transertion. If the processes of transcription, translation, and membrane insertion occur simultaneously at any point in the biogenesis of a cytoplasmic-membrane protein, then the chromosomal locus encoding that protein is transiently tethered to the membrane. Only one polysome is shown for clarity.

**Fig. 2:**
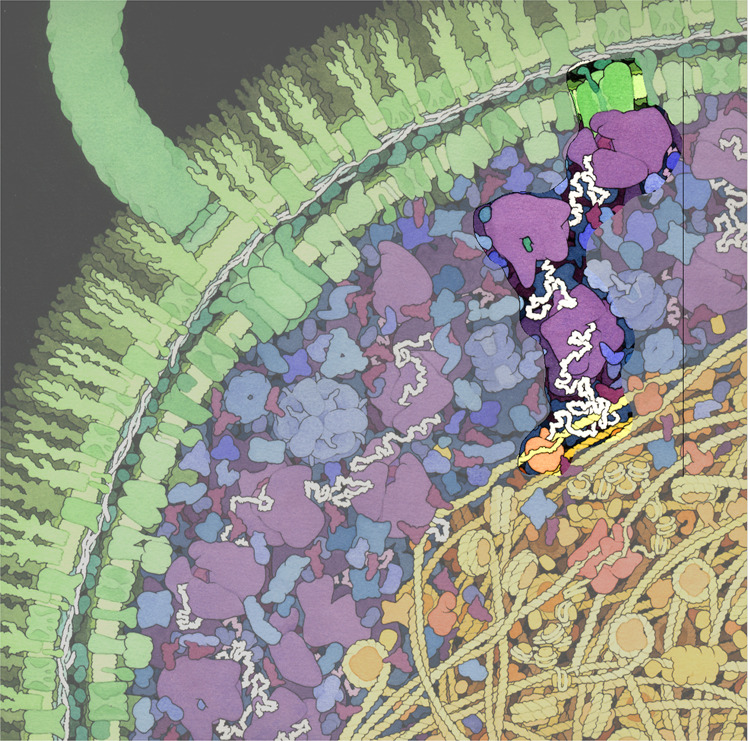
Cross-section of a small portion of an *Escherichia coli* cell. The outer and inner membranes studded with transmembrane proteins are shown in green. The cytoplasmic area is colored blue and purple. Enzymes are shown in blue. The nucleoid region is shown in yellow and orange. An active RNA polymerase and a nascent mRNA (white strand), loaded with ribosomes (purple) and ending at a Sec translocon (green) in the inner membrane, are highlighted as an example of transertion. Original illustration by David S. Goodsell, The Scripps Research Institute (10.2210/rcsb_pdb/goodsell-gallery-001; https://pdb101.rcsb.org/sci-art/goodsell-gallery/escherichia-coli)^33^.

As an alternative to dedicated proteins, chromosome-membrane anchoring through nascent RNA and integral membrane proteins was suggested based on experimental data^10,11^, and described in 1979 by K. Kleppe et al.^15^. The concept was revived in 1995 by V. Norris^16^ who called it ‘transertion’, for simultaneous transcription-translation-insertion of membrane proteins (Figs. 1–2). In parallel, C. Woldringh^17^ considered the same process as a factor in the balance of forces determining the nucleoid morphology. The transertion model was further developed as a hypothesis^18–21^ and examined experimentally^22–27^. The simple fact that the amount of many abundant integral membrane proteins (present in hundreds and thousands of copies) must be doubled during the cell division cycle (as short as 20–25 min) is a reasonable argument for their necessarily simultaneous transcription, translation, and insertion into the membrane. Many of these individually transient but collectively persistent links may well be responsible for the estimated 10–90 attachment sites^11^. The attractiveness of this model is that transertion is an inherent part of the cell metabolism and is a likely result of evolution (see also ref. ^28^).

A set of phenomena consistent with the transertion model includes changes in nucleoid morphology induced by inhibition of RNA or protein synthesis. There are three links in the transertion chain that can be arrested or disrupted: RNA polymerases, ribosomes, and Sec translocons (Fig. 1). If transertion exerts the expansion force on the nucleoid, its disruption should cause a disbalance of forces followed by nucleoid compaction. Indeed, chloramphenicol-induced nucleoid compaction^29^ is a classical phenomenon that inspired the idea of transertion and is thought to occur due to the detachment of stalled polysomes from the gene following transcription run-out^22^. It was demonstrated that these morphological changes are dynamic and caused by an interplay of both membrane anchoring and volume exclusion effects of polysomes and ribosomal subunits, supporting the transertion model^25,26^. The most impressive evidence that this may happen in live bacteria is, so far, the detected reposition towards the membrane of the chromosomal locus encoding a membrane protein upon its expression^24,30^. Localization of RNAs to the membrane in the bacterial cell can be also explained through the transertion mechanism (see ref. ^31^ for a review). Though, as concluded in ref. ^9^, all existing pieces of evidence are indirect.

## Type III secretion system and transertion

T3SS2 of *V. parahaemolyticus* is composed of about 20 different structural proteins and is encoded in an 80-kb chromosomal segment called *V. parahaemolyticus* pathogenicity island (Vp-PAI)^1^. Its main function is the translocation of a dozen of effectors (encoded in the same Vp-PAI) into host cells. Synthesis and assembly of T3SS2 are induced by an external signal (e.g., bile salts) and occur via two-step activation. First, the transmembrane sensor VtrAC activates transcription of *vtrB*, which encodes a second activator. Then, VtrB (also a transmembrane protein) stimulates transcription of multiple structural and effector genes in Vp-PAI, leading to the assembly of the T3SS2 injectisome. Kaval et al.^4^ convincingly demonstrate that: a) the *vtrB* genomic locus is relocated from mid-cell to the membrane upon activation of VtrAC; b) the locus product, VtrB, appears in the membrane adjacently to its locus; and c) the assembled T3SS2 needle is revealed at the same membrane position on the outer side of the envelope. Moreover, even the mRNA of major effector protein VopV (to be injected by T3SS2 into the host cell) is found close to VtrB. These *colocalizations* of activators, genes, and products are interpreted by the authors as their *concurrent* transcription, translation, and membrane insertion—namely, transertion.

The very first step of the induction process—capturing the *vtrB* locus of Vp-PAI by VtrAC—looks like searching for a needle in a haystack. However, the mission is possible based on what is known about other systems: e.g., it takes just about 10 min for the transmembrane pH receptor CadC to bind its distinctive target site on the chromosome of *E. coli*^32^. The membrane location of VtrB in the immediate vicinity of its locus is also not obvious: while nascent proteins are expected to be linked to the gene through transertion, the final VtrB cluster is presumably so large that its diffusion in the membrane may be severely hampered. Furthermore, the length of the 80-kb Vp-PAI would be about 25 μm, suggesting that transertion of all T3SS2 structural components and even effectors in a small membrane area implies high compaction of the island. Alternatively, the membrane domain formed by the primary insertion of VtrB and first structural components may be preferable for the localization of other components as well^21^, promoting self-assembly of the whole complex in-place. Adding a time scale for different steps in the synthesis and assembly of T3SS2 using time-resolved methods may provide a more detailed understanding of this process.

This is indeed the first time that several main components of the transertion complex are found to colocalize. However, the picture is dissimilar from the original transertion model. First, T3SS2 gene promoter(s) are searched by membrane protein activators VtrA and VtrB, in contrast to the relocation of expressed gene loci to the nucleoid periphery (towards the membrane) due to the ‘demixing’ of polysomes. Second, the T3SS2 gene loci are ‘hooked’ to the membrane by VtrAC and VtrB, instead of being anchored through nascent mRNA and proteins to the Sec translocon. The insertion of the inner and outer ring and translocon components of T3SS2 is supposed to be Sec-dependent^1,2^, thus detection of Sec components in the vicinity of VtrB would be consistent with the transertion model. In a more general sense, transertion is thought to be a result of the *constitutive* synthesis of abundant integral membrane proteins, as opposed to the membrane capturing and expression of the entire pathogenicity island *by a demand* upon induction by an external signal. So, why, and how transertion may be efficient for T3SS2 assembly? The T3SS injectisome is made in one or a few copies per cell, and it looks beneficial to bring the whole set of encoding genes to the membrane, express them all at once, and immediately insert the proteins into (or through) the membrane. In this way, a lot of material, energy, and time is saved in contrast to a separate transcription, translation, and further random insertion of T3SS2 components into the membrane.

One last remark (for the youngest readers and not only): while walking along a well-defined track in a forest and being deeply immersed in your current and future tasks (e.g., T3SS), do not miss a strange white rabbit rushing to his hole. Do not hesitate to follow him, and you may discover something very interesting (probably less encouraged, like transertion) that is deeply related to your main interests.
